# Congenital aplasia of the optic chiasm and esophageal atresia: a case report

**DOI:** 10.1186/1752-1947-5-335

**Published:** 2011-08-01

**Authors:** Stefano Pensiero, Paolo Cecchini, Paola Michieletto, Gloria Pelizzo, Maurizio Madonia, Fulvio Parentin

**Affiliations:** 1 Ophthalmology Unit, Department of Surgery, Institute for Maternal and Child Health, Burlo Garofolo Trieste, Via dell'Istria 65/1, I-34100 Trieste, Italy; 2 IRCCS E Medea, Via Cialdini 5, I-33037 Pasian di Prato (UD), Italy; 3 Paediatric Surgery Unit, Department of Surgery, Institute for Maternal and Child Health, Burlo Garofolo Trieste, Via dell'Istria 65/1, I-34100 Trieste, Italy

## Abstract

**Introduction:**

The complete absence of the chiasm (chiasmal aplasia) is a rare clinical condition. Hypoplasia of the optic nerve and congenital nystagmus are almost invariably associated characteristics. Microphthalmos or anophthalmos are common features in chiasmal aplasia, while central nervous system abnormalities are less frequent. Esophageal atresia can be isolated or syndromic. In syndromic cases, it is frequently associated with cardiac, limb, renal or vertebral malformations and anal atresia. More rarely, esophageal atresia can be part of anophthalmia-esophageal-genital syndrome, which comprises anophthalmia or microphthalmia, genital abnormalities, vertebral defects and cerebral malformations. Here, a previously unreported case of chiasmal aplasia presenting without microphthalmos and associated with esophageal atresia is described.

**Case presentation:**

Aplasia of the optic chiasm was identified in a Caucasian Italian 8-month-old boy with esophageal atresia. An ultrasound examination carried out at 21 weeks' gestation revealed polyhydramnios. Intrauterine growth retardation, esophageal atresia and a small atrial-septal defect were subsequently detected at 28 weeks' gestation. Repair of the esophageal atresia was carried out shortly after birth. A jejunostomy was carried out at four months to facilitate enteral feeding. The child was subsequently noted to be visually inattentive and to be neurodevelopmentally delayed. Magnetic resonance imaging revealed chiasmal aplasia. No other midline brain defects were found. His karyotype was normal.

**Conclusion:**

If achiasmia is a spectrum, our patient seems to depict the most severe form, since he appears to have an extremely severe visual impairment. This is in contrast to most of the cases described in the literature, where patients maintain good--or at least useful-- visual function. To the best of our knowledge, the association of optic nerve hypoplasia, complete chiasmal aplasia, esophageal atresia and atrial-septal defect, choanal atresia, hypertelorism and psychomotor retardation has never been described before.

## Introduction

Complete absence of the chiasmal structure, often associated with optic nerve aplasia, is termed chiasmal aplasia, while the term achiasmia is used to identify the abnormality of crossing fibers [1]. In fact, while in albinism the temporal retinal fibers erroneously decussate at the optic chiasm (OC), in achiasmia the majority of fibers fail to cross at the OC and project ipsilaterally. This condition is also termed 'non-decussating retinal-fugal fiber syndrome' (NDRFFS) [2,3]. Both albino and achiasmatic anatomical and developmental abnormalities can be functionally demonstrated by means of flash visual evoked potentials (F-VEPs) [4,5]. In achiasmia, F-VEPs show a higher positive component ipsilateral to the stimulated eye, while in albinos the response is greater contralaterally [4,1].

Congenital nystagmus is a consistent feature in achiasmia, as well as in certain cases of optic nerve hypoplasia [2,6]. Central nervous system (CNS) abnormalities, such as septo-optic dysplasia, hypopituitarism, encephalocele or corpus callosum agenesia are associated with achiasmia [1].

The complete absence of the chiasm (chiasmal aplasia) is a rarer clinical condition. Hypoplasia of the optic nerve and congenital nystagmus are almost invariably associated characteristics [6,7]. Microphthalmos or anophthalmos are common features in chiasmal aplasia, while CNS abnormalities are less frequent [6,7].

A case of chiasmal aplasia in a Caucasian baby, associated with esophageal atresia (EA), is here described.

## Case presentation

Polyhydramnios was detected in a 35-year-old primigravida at 21 weeks' gestation. Esophageal atresia was diagnosed at 28 weeks' gestation on the basis of a small stomach and polyhydramnios on an ultrasound examination. Other abnormalities detected included a dilated upper esophageal pouch and an atrial-septal defect. Our patient underwent periodic therapeutic amniocentesis (1500-1900 ml of fluid per procedure) from 28 weeks' gestation for relief of polyhydramnios and to prevent premature onset of labor. A male fetus with a birth weight of 1980 g was delivered by caesarean section at 37 weeks' gestation. Apgar scores were six and eight at one and five minutes respectively. A clinical examination showed type III EA, right choanal atresia, atrial-septal defect, telecanthus and hypertelorism without any obvious strabismus. His mother's history was negative for familial visual anomalies and there was no evidence of maternal infection or drug abuse during pregnancy. His karyotype was normal.

Uncomplicated esophageal anastomosis with closure of the tracheo-esophageal fistula was performed on the second day of his life. Due to the persistence of suction weakness, our patient was discharged two months later with a naso-gastric tube and enteral nutrition. The baby was referred for pediatric consultation at four months of age. A failure to thrive (length and weight, head circumference on third centile) due to gastroesophageal reflux was documented. A laparoscopic gastric fundoplication was performed. A jejunostomy was also performed to allow continuation of enteral feeding.

The baby underwent a complete ophthalmic evaluation at the age of four months: our patient showed erratic eye movements and was unable to fixate on a light. Horizontal slow nystagmus was observed. His pupil reactivity to light was absent bilaterally. A slit-lamp biomicroscopy of the anterior segment of both eyes was unremarkable. Funduscopy revealed severe bilateral optic disc hypoplasia and mild tortuosity of the retinal blood vessels. His optic discs appeared greyish and oval. F-VEPs were repeatedly non-recordable bilaterally.

**Figure 1:**
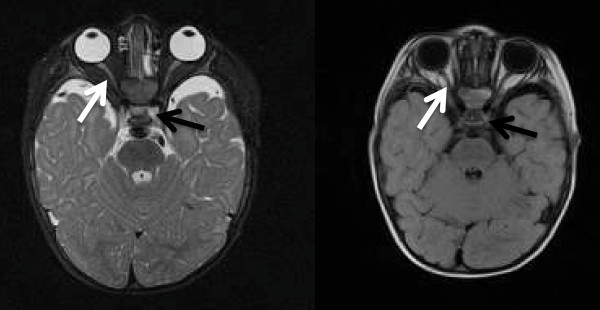
**Axial Tx and Ty MRI scans showing complete absence of the optic chiasm (black arrow); the optic nerves can be identified only in the intraorbital portion (white arrow)**.

Brain magnetic resonance imaging (MRI) scans performed at the age of nine months showed normal eye bulb structures, absence of the OC and optic radiations, consistent with extreme hypoplasia or aplasia of those structures. His optic nerves were bilaterally traced only in the intraorbital portion and were of small appearance. There was no evidence of his optic nerves more posteriorly (Figure 1). His other cerebral structures were normal.

An evaluation at 10 months revealed delayed social skills and language development.

General examination at 18 months of life showed reduced stature, persistence of food aversion, and delayed social contacts and language development.

## Discussion

EA can be isolated or syndromic [8]. In syndromic cases, EA is frequently associated with cardiac, limb, renal and vertebral malformations and anal atresia [8]. In our case, his karyotype was normal, so chromosomal anomalies responsible of syndromic EA (for example trisomy 21, 18, 13 and 17q21.3-q23 deletion) were not involved [8]. Other conditions frequently associated with EA include VACTERL (vertebral anomalies, anal atresia, cardiovascular anomalies, trachea-esophageal fistula, esophageal atresia, renal and/or radial anomalies and limb defects) syndrome, Feingold (oculo-digito-esophago-duodenal) syndrome and Rogers (anophthalmia-esophageal-genital or AEG) syndrome [8]. Other conditions occasionally associated with EA, and characterized by an ocular involvement, are shown in Table 1. Maternal diabetes and phenylketonuria, rarely associated with EA, were excluded due to the normality of blood tests during pregnancy. AEG syndrome comprises EA, anophthalmia or microphthalmia, genital abnormalities, vertebral defects and cerebral malformations [9]. There is evidence of a genetic mechanism for this syndrome. In a number of cases, deletion and/or mutation of the transcriptional regulator gene, *Sox2*, has been proved to be involved [10,11]. The phenotypic variability may be remarkable, ranging from anophthalmia to normal ocular development [12,13]. *Sox2 *also seems to play a causative role in isolated anophthalmia and microphthalmia [11]. However, S *Sox2 *deletion or malfunction can hardly have been involved in our patient, as his eyes were fully developed and the only eye abnormality was the optic nerve hypoplasia. Moreover he presented with no genital or vertebral anomalies, while he did have a heart malformation. He did not present ear anomalies, typical of CHARGE (coloboma of the eye, heart defects, atresia of the choanae, retardation of growth, genital abnormalities, and ear abnormalities) and OAVS (oculo-auriculo-vertebral spectrum) syndromes, but the first is the only syndrome presenting with choanal atresia (Table 1). Our patient could not therefore be classified in any of the syndromes associated with EA.

**Table 1:** Comparison among the clinical characteristic of our case and the syndromic form of EA

Features	Our Case	AEG (Rogers)	VACTERL	Feingold	CHARGE	OAVS	Bartsocas-Papas
**Esophageal atresia**	+	+	+	+	+	+	+

**Microcephaly**	-	+	-	+	+	-	+

**Optic chiasm aplasia**	+	-	-	-	-	-	-

**Nystagmus**	+	-	-	-	-	-	-

**Optic nerve hypoplasia**	+	-	-	-	-	-	-

**Eyes anomalies**	-	+	-	+	+	+	+

**Optic tract aplasia**	+	-	-	-	-	-	-

**Anophthalmia/microphthalmia**	-	+	-	-	-	-	+

**Telecanthus/hypertelorism**	+	-	-	-	-	-	+

**Cerebral malformation**	-	-	-	-	+	-	+

**Heart malformation**	+	-	+	+	+	+	-

**Vertebral defects/other bone anomalies**	-	-	+	+	+	+	+

**Genital/renal anomalies**	-	+	+	+	+	+	+

**Facial/visceral problems**	-	-	-	-	-	+	+

**Visual impairment**	+	-	-	-	-	-	-

**Mental retardation**	+	+	-	+	+	-	-

**Facial cleft**	-	-	-	-	-	-	+

**Limb anomalies**	-	-	+	-	+	-	+

**Ear deformities**	-	-	-	-	+	+	-

**Choanal atresia**	+	-	-	-	+	-	-

In syndromes associated with optic nerve and/or OC hypoaplasia, some genes responsible for the molecular mechanisms of neural routing have been related to the achiasmia spectrum. In the animal model, the lack of the transcription factor Foxd1 induces chiasmal malformation and misprojection of retinal fibers [14]. *Foxd1 *misexpression leads to a cascade of other gene products misregulation, which interferes with the normal development of the OC and with the ratio of ipsilateral to contralateral chiasmal nerve fiber routing [14]. *Foxd1 *has not been described to play a role in EA and it is questionable how it could be involved in the multiple malformations observed in the present report. It seems unlikely to consider EA a merely incidental finding. *Pax *family genes have also been suggested to play an important role in the correct development of the OC [15,16]. In the murine *Pax *mutant model, the OC fails to develop, and retinal axons enter the ipsilateral optic tract [15,16]. Again, the association with EA in our case would be hard to explain.

Sami *et al. *[1] have suggested a classification system for patients affected by achiasmia: type A, presenting with isolated achiasmia and often nystagmus, with possible MRI evidence of a small chiasm; type B, presenting with chiasmal hypoplasia and midline defects (septo-optic dysplasia); and type C, presenting with chiasmal hypoplasia and possible clefting disorders, encephalocele, and agenesia of the corpus callosum. In Sami's series, one patient did not fit into one of the three suggested groups. The child (a boy of six years) suffered from multiple facial, visceral and developmental problems, including EA. An MRI scan showed an isolated small chiasm. The child exhibited horizontal nystagmus. To the best of our knowledge, this is the only previous case of achiasmia in a patient affected by EA, and appears somehow similar to ours: OC aplasia, horizontal nystagmus and esophageal atresia are shared features between Sami's case and ours. However, other traits are remarkably different. The main discrepancy is the presence of the chiasm--even if hypoplasic--in the MRI scans in Sami's report, while no chiasm structure was detectable on MRI scanning and no facial abnormalities were present in our patient. The F-VEPs results and the lack of fixation and of pupil reactivity to light suggest a severe visual impairment in this child, unlike the Sami case, who exhibited fairly good visual function. Moreover our child showed an atrial septal defect and choanal atresia, which was not been described in the Sami case.

One could speculate that these two cases may have the same etiology with different phenotypes. The rarity of this condition and the relevant differences between the two cases, however, suggest great caution in attempting to group them in a single clinical entity.

If achiasmia is a spectrum, our child seemed to depict the most severe form, since he appeared to have an extremely severe visual impairment, in contrast to most of the cases described in literature that maintain a good--or at least useful--visual function. The lack of fixation and reactivity to light or structured stimuli and the presence of roving eye movements were highly suggestive of poor or no residual visual function. The lack of F-VEP response--which is very unusual in achiasmia--confirmed the OC aplasia suggested by MRI findings; moreover we could consider the OC aplasia of our patient to be secondary to a primary bilateral severe optic nerve hypoplasia. Pomeranz [17] described an 18-month-old boy with bilateral optic nerve hypoplasia and OC not identifiable at the MRI who showed profoundly abnormal F-VEP in his right eye. However, left eye stimulation demonstrated a typical VEP occipital asymmetry of the response, consistent with NDRFFS. VEP results showed the presence of a hypoplasic and not aplasic OC (OC was not detectable using MRI scans), with the characteristics of achiasmia. On MRI scans no other major brain abnormalities were detected. In contrast to our case, no other visceral malformations were noticed, and the baby showed a good visual interaction with the environment.

Finally, OC aplasia has often been described in association with other major CNS abnormalities and unilateral or bilateral anophthalmos or microphthalmos was always present. This is in contrast to our case. The genes that are involved in EA and in achiasmia are all located in different chromosomes, so it is difficult to predict simultaneous involvement of multiple genes. In fact *Pax *is located in chromosome 11p13, *Foxd1*1 on 5q12-13, *Mycn *(producing Feingold syndrome) on 2p24.1, *Chd7 *(CHARGE) on 8q12.2 and *Sox2 *(AEG) on 3q26. For this reason the syndrome here described, like VACTERL and OAVS, is probably to be considered of malformative origin.

## Conclusion

We believe that our child does not fit into one of the previously reported achiasmia or OC aplasia types or reports. To the best of our knowledge, the association of optic nerve hypoplasia, chiasmal and optic tracts aplasia (confirmed by VEP and light pupil reactivity absence), with telecanthus and/or hypertelorism, esophageal atresia, atrial septal defect, choanal atresia, and developmental and language delays has never been described before.

## Abbreviations

AEG: anophthalmia-esophageal-genital; CNS: central nervous system; EA: esophageal atresia; F-VEPs: flash visual evoked potentials; MRI: magnetic resonance imaging; NDRFFS: non-decussating retinal fugal fiber syndrome; OC: optic chiasm.

## Consent

Written informed consent was obtained from the parents of the patient for publication of this case report and any accompanying images. A copy of the written consent is available for review by the Editor-in-Chief of this journal.

## Competing interests

The authors declare that they have no competing interests.

## Authors' contributions

SP was a major contributor in writing the manuscript. GP performed surgical intervention.

PC and PM performed clinical and instrumental examinations. MM and FP made a review of the literature and were involved in the diagnosis and management of the patient. All authors have read and approved the final manuscript.
